# The maternal gut–fetal brain axis: role of maternal microbial metabolome in early neurodevelopment

**DOI:** 10.3389/fnut.2026.1903574

**Published:** 2026-09-01

**Authors:** Marianne J. Scoglio, Trinayani Samala, Nasrin Jangjoo Ghalat, Anusha Jayaraman, Sven Pettersson

**Affiliations:** 1 École Polytechnique Fédérale de Lausanne (EPFL), Lausanne, Switzerland; 2 ASEAN Microbiome Nutrition Centre, National Neuroscience Institute, Singapore, Singapore; 3 Department of Dental Medicine, Karolinska Institutet, Stockholm, Sweden

**Keywords:** fetus, gut-brain axis, maternal metabolome, mental health, microbiome, neurodevelopment, nutrition, pregnancy

## Abstract

Fetal neurodevelopment is a critical phase modulated by the maternal microbiome. While certain specific signaling pathways have been identified, the complete signaling network and the spatial and temporal complexity remain largely unknown. Maternal metabolites are essential for fetal growth and certain metabolites are subject to dynamic adaptation during pregnancy. Alteration of maternal microbe richness and diversity, caused by malnutrition, psycho-social distress, and or infection, are cues known to impair fetal neural developmental circuits concomitant with increased risk of cognitive dysfunction later in life. This mini review focuses on the role of maternal microbial metabolites in shaping fetal neurodevelopment.

## 1 Introduction

Maternal nutrition is a critical factor for fetal health and development (1), guided by maternal inputs delivered through the placenta and the umbilical cord. The Developmental Origins of Health and Disease (DOHaD) framework posits that early-life environmental exposures, including the prenatal period, can induce long-term changes in health and disease risk (2, 3). Perturbations such as maternal diet and psychological distress can cause developmental adaptations via fetal programming resulting in permanent structural, physiological, and or metabolic changes that increase disease susceptibility later in life (4). Epidemiological and animal model studies link nutritional deficiencies during pregnancy to elevated risks of diabetes, obesity, stroke, and cardiovascular disease (5), as well as neurodevelopmental disorders including autism spectrum disorder (ASD), attention deficit hyperactivity disorder, and schizophrenia (6–8). Additionally, exposure to prenatal maternal stress exerts wide-ranging effects on brain development, including alterations in regional brain volumetric growth, cortical folding, and functional connectivity, associated with long-term cognitive, language, and behavioral impairments (9–13).

The gut microbiome influences immune function, digestion, metabolism, and brain activity through the gut–brain axis (14), with gut microbes producing a range of metabolites that signal by directly crossing the cell membrane (hydrophobic compounds) or through ligand–receptor pathways (15). Gut metabolites can enter the maternal circulation and cross the placenta to influence fetal brain development (16–19). Gestational changes in the gut microbiota and its metabolites, including short-chain fatty acids (SCFAs), remain debated, with studies variously reporting stability or marked compositional shifts (20, 21), potentially reflecting dynamic regulation of only specific metabolites to meet time-sensitive developmental demands. Disruption of the maternal gut microbiome by malnutrition or stress can often alter fetal gene expression, neural circuit development, and potentially long-term brain function and behavior in adulthood (22–26).

**Figure 1:**
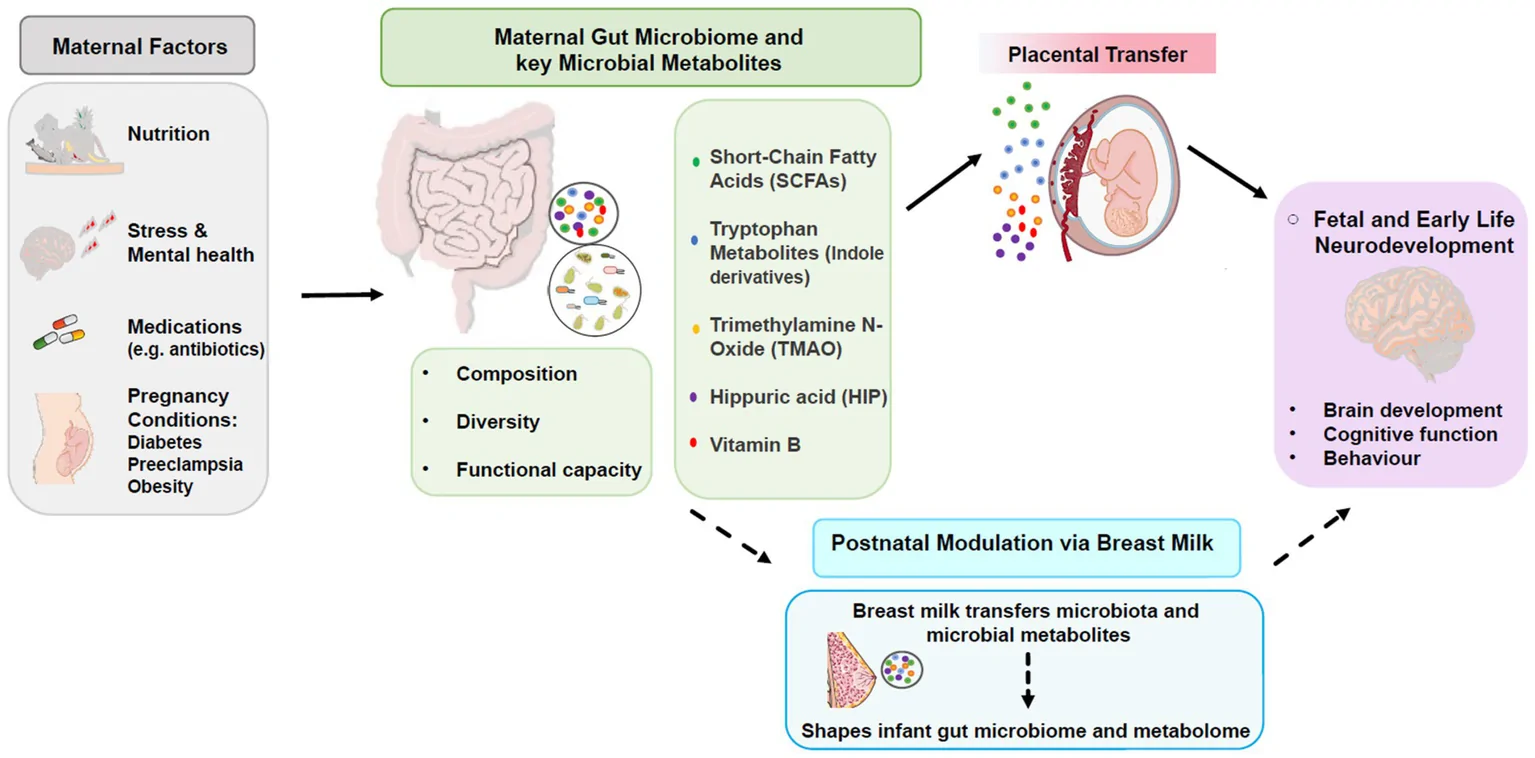
Illustration of maternal gut-fetal brain axis. Maternal factors affect maternal gut microbiome and microbial metabolite profiles, which in turn can influence fetal and early life neurodevelopment through placental transfer during pregnancy and or postnatally through breast milk. This Figure was created using some icons reproduced from NIAID NIH BioArt and Biocons, licensed under CC0 and CC BY 3.0. Some icons were reproduced from Servier Medical Art, licensed under CC BY 3.0 and CC BY 4.0. Some of the icons were adapted using Inkscape (v1.4).

Studies have reported the prenatal intrauterine detection of bacterial DNA and microbial signatures in the placenta, amniotic fluid, and fetal tissues suggesting the possibility of direct maternal-fetal microbial transmission (27–30). However, the findings have raised concerns regarding sample contamination, low bacterial load, and technical artifacts and this concept of intrauterine microbiome remains highly controversial requiring further validation (31–33). Emerging evidence still strongly supports the view that a healthy intrauterine environment during pregnancy is largely sterile, while maternal microbial metabolites can readily cross the placental barrier through circulation and influence fetal development as mentioned before. Therefore, in this mini review, we focus on the more robust experimental and clinical evidence-based indirect effects of changes in the maternal gut microbiota mediated through their metabolites on fetal neurodevelopment: a maternal gut–fetal brain axis (Figure 1).

## 2 Maternal gut microbiome modulates neurodevelopment

Germ-free (GF), antibiotic-treated, and dysbiosis-induced mice are commonly used to achieve controlled microbial alterations to assess their effects on host physiology. Depletion of the maternal microbiome in GF mice influences embryonic microglia transcriptomic and chromatin accessibility levels in a time- and sex-dependent manner (34). Offspring of GF dams exhibit markedly reduced levels of microbial metabolites compared to those of specific pathogen-free (SPF) mice (16). Comparing GF and SPF fetuses via gene expression profiling and untargeted metabolomics revealed a major impact of the maternal microbiota on prenatal gene regulation, with genes involved in interferon and inflammatory signaling, neuronal development, and synaptic function downregulated in GF fetal brains (17). Antibiotic treatment of pregnant mice results in fetuses with altered brain transcriptomic profiles and impaired thalamocortical axonogenesis (23), and by 4 weeks of age their offspring show reduced hippocampal CA1 pyramidal neuron numbers, synaptic density, and astrocytic surface area, alongside hypomyelination of the corpus callosum, decreased dentate gyrus neurogenesis, and impaired spatial learning and memory (35).

Human studies, in contrast, are largely observational and longitudinal, correlating either maternal microbiome composition during pregnancy, and or impact of breast milk and its composition with offspring neurodevelopmental outcomes and innate and adaptive immune system after birth (36–39). Pregnant women with gestational disorders such as preeclampsia show disrupted microbial composition (40), with several microbial taxa exhibiting significant causal associations with the condition (41). This is further correlated with adverse neurological outcomes in offspring in both clinical and animal model studies (42). High anxiety and stress in mothers resulted in reduced alpha diversity of infant microbiome and reduced relative abundance of beneficial bacterial species associated with gut-brain axis and immune function regulation compared to those from mothers reporting less anxiety and stress (43).

Experimental induction of gut dysbiosis during pregnancy can result in adverse outcomes similar to that of preeclampsia (44). The microbiome also shapes the offspring immune system, including induction of TH17 cells that produce IL-17a (45). Aberrant microbe-immune communications or external manipulation of microbe composition has been shown to increase risk of ASD related problems, intestinal and neuronal inflammation as well as cognitive problems related to anxiety and repetitive disorders (45–48). Maternal stress modulates the maternal gut microbiota, which can be vertically transmitted to offspring, influencing offspring behavior in mice (49). Seven bacterial taxa were identified in a rat model as biomarkers for cross-generational depression, characterized by their shared enrichment in both prenatally stressed dams and their offspring. These taxa were also strongly correlated with altered microbial metabolic pathways in offspring, including glycine, serine, threonine, and glycerophospholipid metabolism, frequently implicated in depression. Moreover, stress-induced microbiome associated alterations in glycine, serine, and threonine metabolism were strongly correlated with increased neuroinflammation and neurotransmitter imbalances in the prefrontal cortex of offspring (49). Collectively, converging evidence from these studies highlights the critical importance of the maternal gut microbial environment for fetal neurodevelopment and illustrate the importance of harboring functional microbiota richness and diversity.

## 3 Maternal diet-metabolome effects on the fetal brain

Alterations in maternal nutrition, such as excess sugary food and over-processed food consumption can result in alteration of the maternal microbiome, and are associated with behavioral and cognitive problems in offspring (50, 51). A recent study linked higher third-trimester total cholesterol to lower offspring birth weight and reduced cord blood indole levels (52). In mice, deficits of protein intake during pregnancy induce extensive impairment of fetal brain transcriptomic and metabolomic profiles resulting in cognitive and anxiety-like behavioral patterns in adult offspring (53).

Metabolites such as SCFAs influence fetal neurodevelopment through epigenetic regulation, ligand-receptor mediated signaling pathways, and metabolic regulation, ensuring correct temporal and spatial progression of neurodevelopment (54). Intake of a Western diet rich in sugar and fat content during pregnancy and lactation in piglets has been shown to affect SCFA levels in sows and young piglets and is correlated with decreased neurogenesis in piglets (55). In mice, a maternal low-fiber diet during gestation led to impairment of cognitive function and synaptic plasticity in offspring, which was reversed by butyrate supplementation, but not propionate (56). A maternal high-fat diet in mice reduces the relative abundance of SCFA-producing gut bacteria, decreasing maternal cecal butyrate levels and impairing intestinal barrier integrity. These alterations are associated with placental hypoxia, reduced vascularization, and changes in the placental metabolomic profiles, notably lower carnitine levels (57). This is significant given carnitine’s neuroprotective role in mitochondrial fatty acid metabolism and the association of carnitine deficiency with ASD (58, 59). The SCFA, propionate, separately regulates embryonic sympathetic nervous system development via GPR41 signaling, promoting neuronal differentiation; propionate deprivation leads to sympathetic dysfunction in GF offspring, while maternal high-fiber diet and propionate supplementation rescue delayed sympathetic cardiac innervation (60). In human studies, unbalanced diet-associated disruption of the maternal gut microbiome can dysregulate multiple SCFAs that modulate blood pressure through GPR41 activation, with isobutyric, propionic, and acetic acids reported significantly elevated in preeclampsia patients (61). A high-fiber diet or SCFA supplementation restored maternal obesity-induced microbial dysbiosis and cognitive and social dysfunctions in offspring (62). Butyrate-producing bacteria were enriched in maternal fecal samples at the third trimester from mothers of children with normative behavior compared to mothers of children with internalizing behavior at 2 years of age (63).

Notably, elevated levels of trimethylamine N-oxide (TMAO), a gut microbiota–derived metabolite, were associated with preeclampsia (64). Elevated TMAO has been implicated in accelerated atherosclerosis and may contribute to preeclampsia pathophysiology (61). The neurodevelopmental effects of TMAO remain controversial: In mice, elevated postnatal levels are associated with neuropathy, cognitive impairment, and impaired hippocampal neuronal function, whereas prenatal exposure is linked to increased thalamocortical axonogenesis (23, 65). Choline is recycled and requires regular supplementation through diet (66). Choline is a major methyl donor supporting one-carbon metabolism and DNA methylation, which regulate gene expression during neurogenesis and cell differentiation (67, 68). Disruption of one-carbon metabolism increases the risk of embryonic neural tube defects and adversely affects long-term cognitive and behavioral outcomes (69–71). Gut bacteria compete with the host for dietary choline, converting it to TMAO (71), and because both choline and TMAO appear to influence neurodevelopment, inter-individual differences in microbial choline metabolism should be considered when defining optimal maternal nutrient requirements (64).

Maternal tryptophan levels fluctuate during pregnancy, which can alter the psychosocial development of offspring by reprogramming their neuroendocrine system and gut microbiome (72). Tryptophan is an essential amino acid obtained only through diet and gut microbiota metabolize tryptophan into indole and indole derivatives (73). Tryptophan and its microbe mediated metabolites play a critical role in tuning anxiety in mice (74). Recent studies demonstrate that exposing GF mice to indole reduces anxiety via SK2 channels in the basolateral amygdala (74). The relevance of this indole effect in humans remains to be confirmed.

Embryos from antibiotic-treated and GF dams exhibit reduced axonogenesis gene expression and deficient thalamocortical axons (23). Supplementation of antibiotic-treated dams with selected microbial metabolites—TMAO, 5-aminovaleric acid, indolepropionic acid (IP), and hippuric acid (HIP)—rescued these defects and significantly increased fetal brain levels of TMAO and IP, suggesting these metabolites in particular mediate thalamocortical axonogenesis *in vivo* (23). In contrast, SCFA supplementation did not significantly affect fetal thalamocortical development (23). Several microbial metabolites, including HIP and IP, correlated with the expression levels of genes involved in interferon and inflammatory signaling, neuronal development, and synaptic function, with more differentially regulated genes in male than female fetuses (17). Taken together, these studies highlight the role of maternal gestational diet and corresponding changes in the microbial metabolite profiles on fetal neurodevelopment.

Thiamine (Vitamin B1) is synthesized only by plants and microorganisms, including our gut microbiota. Although gut bacteria and fungi are a source of thiamine, for human beings, these amounts are not sufficient and thiamine is predominantly supplied by diet (75). Thiamine is very important for maintaining the functions of the nervous system and supporting the synthesis of myelin and neurotransmitters like acetylcholine, serotonin, and glutamate (76). Thiamine deficiency during gestation is associated with poor nutritional status and adverse perinatal outcomes and can impact both mothers and fetuses (77–79). Human studies have shown that high-infant mortality, intrauterine growth retardation, and cognitive damage in children have been associated with thiamine deficiency during pregnancy (80–82), while thiamine supplementation has been shown to have beneficial effects on both prenatal and postnatal outcomes (83, 84). In animal models, maternal thiamine deficiency has been shown to reduce thiamine-dependent enzyme activities in the cerebral cortex, cerebellum, and brainstem of offspring with long-term effects (85, 86), decreased the nuclear density and nuclear size of fetal hippocampal neurons (87), impaired behavior and decreased neurotransmitter levels (88). Recent evidence in pigs has shown that microbe-derived thiamine can activate placental Notch signaling to regulate angiogenesis and nutrient transport in the fetus and increase SCFA production, thus improving maternal-fetal health during pregnancy (89). These studies suggest that maternal diet-derived and gut microbe-derived thiamine plays a key role in offspring growth and neurodevelopment.

## 4 Breast milk and early-life neurodevelopment

Early infancy represents another critical window for neurodevelopment. Human milk, recognized by the World Health Organization as the optimal source of infant nutrition, has been shown to exert lasting effects on brain structure and function (90). After birth, gut-derived metabolites continue to shape brain development through two main pathways. First, a limited number of studies have identified maternal gut microbial metabolites in human milk, suggesting direct transfer from the maternal circulation to the infant (90–92). Second, and more extensively studied, human milk composition shapes the infant gut microbiome, which in turn produces metabolites that influence neurodevelopment (31, 92). The composition of infant formula differs from that of human milk and is associated with distinct metabolite and microbiome profiles, potentially leading to different neurodevelopmental outcomes (93). Maternal feces, breast milk, and neonatal feces have been shown to share several obligate anerobes such as *Bifidobacterium, Bacteroides, Parabacteroides* and members of the *Clostridia,* suggesting vertical transfer of maternal gut microbiota through breast milk (31). Breast milk microbiota has been shown to change within a feeding session and over time, thus directly affecting the offspring gut microbiota composition (94). Nicotine exposure through breast milk induced persistent gut dysbiosis in offspring, with sex-specific outcomes in eating patterns and adiposity (95).

SCFAs have been detected in human milk and cow milk, although their concentrations vary between individuals (96). SCFAs produced in the infant gut have been implicated as mediators of breast milk effects on brain development. Higher SCFA levels during early infancy have been associated with less favorable neurodevelopmental outcomes, suggesting that their effects may be highly dependent on concentration and developmental timing (31). Breastfed infants exhibit lower and more stable fecal SCFA concentrations during the first months of life compared with mixed-fed infants (97). In addition, human milk is an exclusive source of tryptophan for breastfed infants. Although indole derivatives (metabolized from dietary tryptophan by gut microbiota) have not been detected directly in human milk, breastfed infants show significantly higher production of indole-3-lactic acid and indole-3-acetic acid by their gut microbiota, with elevated levels detected in stool compared with formula-fed infants (98). Additionally, elevated levels of sulfated bile acids were present in stool samples of infants given human milk compared to increased secondary bile acids in formula-fed babies (93). However, this study showed that the microbial metabolite profiles of both breastfed and formula-fed infants converged when solid food was introduced around 12 months of age (93). Another study showed that human milk enhanced the production of several lactobacilli-mediated metabolites such as hydroxyphenyllactic acid, tyrosine, indoles, vaccenic acid, and di-peptides, whereas formula enhanced several unique metabolites such as isonicotinic acid and riboflavin (99). Although the significance of these metabolite changes for offspring neurodevelopment is not completely understood, together, these findings open opportunities for early strategies through monitoring and optimizing human milk and formula composition.

## 5 Conclusions and future directions

Gestation represents a critical window of neurodevelopment with long-lasting consequences throughout lifespan. Growing evidence, presented here, illustrates the importance of the maternal microbes and metabolites pivotal for the fetal brain axis. Likewise, diet and environmental stress factors are known to affect maternal microbiome richness and diversity thus further guiding fetal neurodevelopment and, later postnatal growth and brain function. This mini review serves its purpose by becoming the foundation of further microbe-brain studies relevant both pre- and early postnatal maternal and fetal interactions. However, there are limitations to consider: the studies presented in this review heavily rely on rodent models and lack long-term follow-up of offspring, thus restricting the interpretation and scope of the findings. Furthermore, postnatal neurodevelopment is considerably different between mice and humans adding to limitation of the interpretations. Finally, the review focuses only on a subset of the most extensively studied gut microbial metabolites, but additional microbe metabolites remain to be evaluated. Future research should include large-scale longitudinal studies to assess gestational effects on fetal neurodevelopment as well as on neuropsychiatric parameters into adulthood of offspring and to assess microbial metabolites as early biomarkers of neurodevelopmental risk. Preventive and therapeutic studies should evaluate microbial metabolite modulation/supplementation, probiotics, and dietary interventions on maternal prenatal and mental health and microbiome to improve neurodevelopmental outcomes in children. As our understanding of the maternal gut-fetal brain axis continues to evolve, future clinical strategies that promote optimal neurodevelopmental outcomes across the lifespan will emerge.
